# Outbreak of Skin Infections Due to Panton-Valentine Leukocidin-Positive Methicillin-Susceptible Staphylococcus aureus in a French Prison in 2010-2011

**DOI:** 10.1371/currents.outbreaks.e4df88f057fc49e2560a235e0f8f9fea

**Published:** 2014-03-07

**Authors:** Céline Bourigault, Stéphane Corvec, Virginie Brulet, Pierre-Yves Robert, Olivier Mounoury, Chloé Goubin, David Boutoille, Bruno Hubert, Michèle Bes, Anne Tristan, Jérôme Etienne, Didier Lepelletier

**Affiliations:** Bacteriology and Hygiene Department, Nantes University Hospital, 44000 Nantes, France; Bacteriology and Hygiene Department, Nantes University Hospital, 44000 Nantes, France; University of Nantes, EA 3826, Medical School, 44000 Nantes, France; Jail Medical Center, 44000 Nantes, France; Jail Medical Center, 44000 Nantes, France; Jail Medical Center, 44000 Nantes, France; Jail Medical Center, 44000 Nantes, France; Infectious Diseases Department, Nantes University Hospital, 44000 Nantes, France; Regional Unit of Epidemiology and Diseases Control, Pays de la Loire Area, 44000 Nantes, France; National Reference Center for Staphylococci, University of Lyon, 69000 Lyon, France; National Reference Center for Staphylococci, University of Lyon, 69000 Lyon, France; National Reference Center for Staphylococci, University of Lyon, 69000 Lyon, France; Bacteriology and Hygiene Department, Nantes University Hospital, 44000 Nantes, France; University of Nantes, EA 3826, Medical School, 44000 Nantes, France

## Abstract

Background. An outbreak of PVL-positive MSSA skin and soft tissue-infections (SSTIs) was suspected in May 2010 when recurrent SSTI was diagnosed in an inmate of a large prison in Nantes, France. Methods and findings. Retrospective and prospective investigations were performed. Microbiological characterisation was by DNA microarray testing (S. aureus genotyping - Identibac, Alere). We identified 14 inmates meeting our clinical and microbiological case definition for PVL-MSSA SSTI between March 2010 and April 2011. The SSTIs developed in tattooed areas in 4 patients and in areas shaved daily with a mechanical razor in 4 other patients. All case isolates exhibited a similar SmaI pulsed-field gel electrophoresis pattern. Microarray analysis showed that all 14 isolates harboured genes encoding PVL and enterotoxins (A, H, K, and Q) and belonged to clonal complex 1 (CC1). Individual and collective hygiene measures, education delivered to inmates and prison employees, and antibiotic treatment of SSTIs were successful in controlling the outbreak. No new cases were identified after April 2011. Routine screening for PVL-positive MSSA carriage was not feasible. Conclusions. Our data suggest that tattooing and shaving with mechanical razors may constitute risk factors for SSTIs among previously colonised inmates and contribute to the PVL-MSSA outbreak in the prison. Allowing inmates access to professional tattooists and to the hygiene and safety conditions available to people in the community would help to prevent tattoo-related infections.

## Introduction

*Staphylococcus aureus *commonly resides as a commensal on human skin and mucous membranes. Thus, asymptomatic cutaneous and nasal ***S. aureus ***carriage is found in about one-third of healthy individuals.^1 ^However, *S. aureus* can cause infections ranging from minor skin and soft-tissue infections (SSTIs) to life-threatening pneumonia or toxin-mediated diseases. Panton-Valentine leukocidin (PVL) production by *S. aureus* is associated with both SSTIs and severe infections such as necrotizing pneumonia. 2 ^,^ 3 ^,^ 4 PVL can be produced by both methicillin-susceptible S. aureus (MSSA) and methicillin-resistant *S. aureus* (MRSA) strains.^2^^,^ 5 PVL-producing *S. aureus* strains are typically acquired in the community by healthy young children or adults. Community-acquired infections with PVL-producing *S. aureus*, most notably MRSA strains, have been documented in a broad range of defined populations such as children, native Americans, members of athletic teams, military recruits, and prison inmates.^6^^,^ 7 ^,^ 8 ^,^ 9 Here, we report the investigation of a PVL-MSSA outbreak in a prison, as well as the measures that were successful in stopping the outbreak. In May 2010, a PVL-MSSA strain was identified at the Nantes University Hospital laboratory in an inmate of the Nantes prison evaluated for recurrent SSTI. The infection-control team was alerted when a laboratory database search identified additional cases. An investigation was carried out jointly with the local and national health authorities and the prison authorities. Several measures were then taken to control the outbreak.****

## Methods

**Setting**. The prison is in Nantes, a city of north-western France with a population of about 500,000. Most of the 440 male inmates serve lengthy terms. There are five cellblocks (A, B, C, D, E), including a maximum-security section, and 330 employees. In the prison, a medical centre that is an outreach component of the Nantes University Hospital emergency department provides outpatient care to inmates. Microbiological samples from inmates are sent to the bacteriology and hygiene department of the Nantes University Hospital. The inmates have daily free access to common areas (e.g., gym, library, and common room) and share bathrooms. Meals must be taken in the cells.

**Definitions of cases and outbreak period.** We used a clinical and microbiological definition to describe the outbreak. The index case was an inmate who had an SSTI in early 2010 followed by a recurrence in May 2010. This recurrence prompted tests for the PVL toxin. The laboratory database was searched to identify all **S. aureus **isolates from soft-tissue lesions sampled between January 2006 and June 2010. Inmates with PVL-positive MSSA SSTIs during the study period were defined as cases; inmates with PVL-negative MSSA SSTIs were not cases.The outbreak period was defined as the period between the first and last PVL-positive MSSA SSTIs.********

**Prospective clinical and microbiological surveillance.** Starting in July 2010, all inmates with suspected SSTIs were encouraged to seek advice from the prison healthcare professionals. Microbiological documentation of all SSTIs was obtained via clinical sample collection for PVL-MSSA testing. For each inmate with a PVL-positive MSSA SSTI, a prison healthcare professional completed a questionnaire to collect clinical and epidemiological data. The number of inmates without SSTIs and prison employees was too large to allow routine screening for PVL-MSSA colonisation.

**Evaluation of high-risk behaviours in inmates. **Exposures were initially evaluated among inmates during the medical consultation for the management of SSTIs. In a more general way, high-risk behaviours were evaluated in a specific survey among all inmates who presented at the prison medical center in March 2011. The interviews were conducted by prison healthcare professionals after informed consent of the inmates. The prevalences of tattooing and body shaving with razors were estimated. We decided not to evaluate sexual practices and drug taking behaviours.

**Microbiology**. Swabs of the SSTI lesions were incubated overnight on blood agar (bioMérieux, Marcy-l'Etoile, France) at 37°C. Suspected colonies were confirmed as *S. aureus *using PastorexTM Staph-Plus (BioRad, Marnes-la-coquette, France). PVL screening was by polymerase chain reaction (PCR) and confirmation testing was performed by the French National Reference Centre for Staphylococci.^10^^,^ 11 Genotyping of each isolate was achieved using a DNA microarray test (*S. aureus *genotyping – Identibac; Alere Technologies GmbH, Jena, Germany) according to the manufacturer’s instructions. Relatedness among all *S. aureus* isolates was investigated by pulsed-field gel electrophoresis analysis using SmaI digestion, as described elsewhere,^10^ and was interpreted according to Tenover et al.^12^ Pulsed-field gel electrophoresis profiles were analysed using Bionumerics software (Applied Maths, Sint-Martens-Latem, Belgium).

**Infection control program**. The inmates received instruction about the benefits of frequent hand washing, regular but not excessive bathing/showering, not sharing personal items (e.g., towels and razors), and cleaning their cells frequently. Both the inmates and the employees received information on helpful behaviours such as placing a towel or item of clothing between the skin and shared equipment in collective facilities (e.g., the gym), cleaning shared facilities and equipment frequently with disinfectant products (only detergents were used before the outbreak), use by employees of alcohol hand rubs (inmates were not allowed to use products containing alcohol), and banning patients with active SSTIs from the gym. Flyers providing information on infection control were distributed throughout the prison and posters were put up in all five cellblocks and in the library and cafeteria. Each SSTI patient was given specific guidelines on minimising the risk of dissemination.

**Management of SSTIs. **Inmates with SSTIs were given oral amoxicillin-clavulanate, 3 g/day for 10 days. They took the antibiotic themselves in their cells. Incision and drainage were performed if needed. The lesions were covered with clean dry dressings. For nasal MSSA decolonisation, mupirocin was applied to the nares twice a day for 5 consecutive days starting 1 week after SSTI resolution. In addition, the patients showered daily with chlorhexidine soap. Recurrent SSTIs were managed with oral clindamycin, 1.8 g/day for 10 days, and nasal decolonisation. After nasal decolonisation, routine screening for nasal MSSA carriage was not performed.

**Ethics statement**. The study has been approved by the French Ministries of Health and of Justice and by the National Health Institute. The methodology of the investigation in the jail was also approved by the National Department of Health Emergency. Systematic oral consent was obtained from prisoners and archived in all medical records.

## Results

**Outbreak investigation and characteristics of patients and SSTIs.** The index case was identified in late May 2010 when he was diagnosed with recurrent SSTI due to a PVL-positive MSSA. This inmate was incarcerated in November 2006. He had a facial abscess in February 2010 followed, in May 2010, by a subcutaneous abscess of the right armpit, at the site of a cut caused by a razor. He tried to drain both abscesses by puncturing them with flamed needles. He had inflammatory psoriasis with multiple excoriations. The retrospective database search identified three further PVL-positive and six PVL-negative MSSA strains recovered from soft-tissue lesions between January 2006 and June 2010 (Figure 1). Prospective surveillance identified ten additional PVL-positive MSSA SSTIs between July 2010 and April 2011 (Figure 1). No new cases were detected after April 2011; after this date, all SSTIs were caused by sporadic PVL-negative MSSA strains.The characteristics of the 14 cases are detailed in Table 1. Median age was 41 years (inter-quartile range [IQR], 33-43). Median time from incarceration to the first PVL-positive MSSA culture was 48 months (IQR, 17-82). The 14 patients represented all five cellblocks. Of the 6 patients with recent tattoos, 4 developed SSTIs in the tattoo area within the following 3 weeks. The 4 patients who shaved their body daily with mechanical razors had SSTIs in the shaved areas. Two other patients had SSTIs in the vicinity of previous skin lesions (psoriasis and vascular ulcer, respectively). Finally, two SSTIs occurred in the absence of previous skin lesions and initially resembled spider bites. Five patients experienced recurrences within a mean of 60 days (36-87 days) after initial antibiotic treatment and nasal decolonisation. Five patients were released from the prison during the outbreak. No prison employees experienced SSTIs during the investigation.

**Microbiological analysis.** All 14 case isolates exhibited a similar*SmaI*pulsed-field gel electrophoresis pattern (Figure 2). Microarray analysis showed that all 14 isolates harboured genes encoding PVL and enterotoxins (A, H, K, and Q) and belonged to clonal complex 1 (CC1). The 10 SSTIs diagnosed before and after the outbreak were caused by various PVL-negative MSSA strains (Figure 2).

**Evaluation of high-risk behaviours in inmates. **Of the 79 inmates seen at the prison medical centre in March 2011, 14 (18%) had tattoos performed during the outbreak (median per inmate, 4; range, 1-11) and 29 (37%) reported shaving their body with mechanical razors during the outbreak. All the tattoos were performed by the same 3 inmates. As French law prohibits tattooing in prisons, we were unable to interview these 3 inmates to evaluate their hygiene practices and equipment.

**Epidemic curve of a PVL-positive methicillin-susceptible (MSSA) skin and soft-tissue infection (SSTI) outbreak in a French prison.**
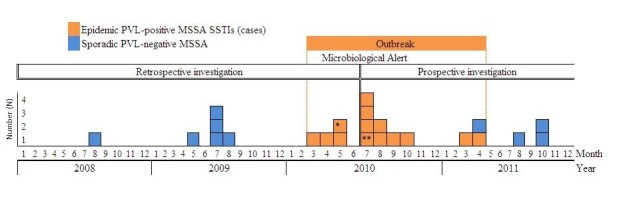
*Index case **Inmate believed to have introduced the epidemic strain in the prison Microbiological alert : the infection control team was alerted by the bacteriology laboratory following the repeated isolation of PVL-positive MSSA isolates. The x axis represents the date of PVL-toxin MSSA isolation from SSTIs.

**Banding patterns determined by pulsed-field electrophoresis (PFGE) and dendrogram showing the genetic relatedness of 24 MSSA isolates recovered from inmates of the Nantes prison, France, in 2008-2011 (numbers 1 to 6 and 21 to 24: PVL-negative MSSA non-epidemic strains; numbers 7 to 20: PVL-positive MSSA epidemic strain).**
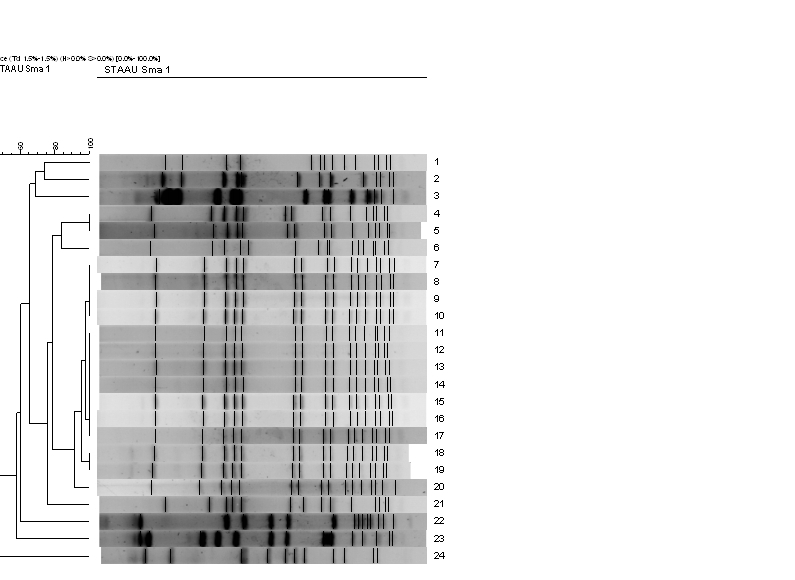

**Figure:**
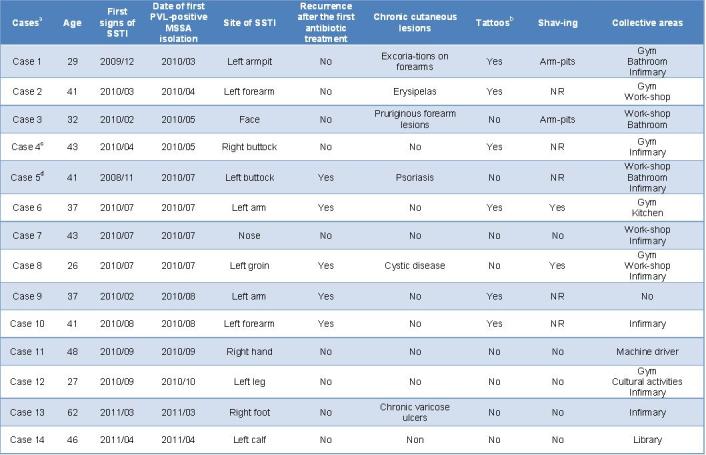
**Table 1. Characteristics of the 14 inmates who experienced PVL-positive MSSA skin and soft-tissue infections during the outbreak at the Nantes prison, France, in 2010-2011.**

## Discussion

We report a fully investigated and controlled SSTI outbreak in a French prison. To our knowledge, no other outbreaks of PVL-positive MSSA SSTIs have been reported in European correctional-facility inmates.

We identified 14 cases of PVL-positive MSSA SSTIs. The actual number may be higher, since SSTIs were not sampled routinely before the introduction of the prospective clinical surveillance programme. Some inmates with SSTIs may have failed to seek assistance from the prison healthcare professionals, and others may have had SSTIs that were not sampled. The patient believed to have introduced the epidemic strain into the prison was incarcerated in 2008. We do not know whether he was colonised with this strain at the time. He had chronic inflammatory psoriasis but waited several months before seeking help from the prison healthcare professionals. His history of SSTI was identified during the outbreak, in July 2010, when he presented to the prison medical centre. His first skin abscess occurred in November 2008. He subsequently had skin abscesses or boils in February 2010 (abdomen), March 2010 (around the navel), April 2010 (sacrum), and July 2010 (abdomen and forearm). In July 2010, the lesions were incised and drained and samples were sent to the microbiological laboratory. The epidemic strain probably spread by skin-to-skin transmission, since the 3 patients identified in March and April 2010 were incarcerated in adjoining cells on the same floor of the same cellblock. All 3 patients worked in the collective areas, which may explain the spread of the epidemic strain to inmates from other cellblocks; isolating these 3 patients in their cells was deemed unethical, particularly as they were paid for their work. Furthermore, some of the inmates had activities associated with a risk of injuries (e.g., carpentry, cooking, or machining solid objects) that were potential portals of entry for micro-organisms. However, some of the SSTIs developed in healthy areas. Male-to-male sexual activity and intravenous substance abuse are common in prisons and can contribute to the transmission of *S. aureus. *We were unable to document these behaviours during our investigation.

A substantial proportion of the cases had tattoos or shaved their body with mechanical razors during the outbreak, suggesting that these practices may have promoted the transmission of *S. aureus* in previously colonised individuals. However, we had no data on colonisation prior to the outbreak. Of the 79 inmates interviewed in March 2011, 29 (37%) reported shaving their body every day with mechanical razors. This prevalence was not significantly higher than in the patients with SSTIs (4/14, 28%). The prevalence of tattooing during the outbreak was lower in the 79 interviewed inmates (14/79, 18%) than in the patients with SSTIs (6/14, 43%), but the difference was not statistically significant. However the sample size and the study design were not sufficient to make detecting a causal relationship between exposure to tatoo and PVL-toxin positive MSSA SSTI. It is simply a concomitant observation and most abscesses occurred in the weeks following a tattooing or skin shaving. That is why we discuss this hypothetis (skin lesion favoring s*staphylococcus *inoculation) without being able to confirm or compare it with other risky practices (sexual practices and the use of intravenous drugs). But the chronology of observations was troubling and the incidence of abscesses significantly decreased following the decrease in the number of observed tattoos and the disappearance of the epidemic strain from April 2011. In a multicentre European study, about 20% of prison inmates had a lifetime history of receiving tattoos while incarcerated.^13^ Our sample sizes were too small to allow a case-control study of risk factors associated with PVL-positive MSSA SSTI; in addition, we had no data on the reservoir, since routine screening for PVL-MSSA colonisation was not feasible. A case-control study of male inmates in a Los Angeles county jail identified several risk factors for MRSA infection, many of which were pre-incarceration factors such as previous skin infection and low level of education;^14^ however, several risk factors were amenable to improvement, including showering frequency, knowledge about Staphylococcus, and soap sharing. Tattooing is known to be associated with infections due to bacteria -- including *S. aureus* -- or viruses.^15^ In correctional facilities, tattooing is performed by the inmates themselves, who generally have no specific training and use makeshift equipment.^16^^,^ 17 The equipment is not sterilised and is frequently shared among inmates. Skin infections related to tattooing usually develop within a few days or weeks. Allowing inmates access to professional tattooists and to the hygiene and safety conditions available to people in the community would help to prevent tattoo-related infections. The French National Authority for Health published new guidelines in 2012 to control the spread of infectious diseases in correctional facilities and recommend allowing professionals to perform tattoos for inmates in prison.^18^

The infection control strategies used during the outbreak were based on guidelines or experience described elsewhere and were adapted to meet local requirements.^19^^,^ 20 They were approved by local health authorities and by the French Ministries of Health and of Justice. These strategies were successful in controlling the spread of the PVL-positive MSSA strain, as no new cases were detected after April 2011. Most PVL-positive outbreaks reported in the US were due to MRSA strains.^21^ No MRSA strains were recovered during the outbreak studied here. However, earlier studies found no significant differences between inmates with MSSA and those with MRSA for age, gender, ethnicity, previous incarcerations, or co-morbidities.^22^^,^ 23 ^,^ 24 ^,^ 25 PVL-positive MSSA and PVL-positive MRSA strains are probably similar regarding their ability to spread and to cause invasive disease in incarcerated populations. During the outbreak, most of the cases had several episodes of PVL-positive MSSA SSTIs, in keeping with data on MRSA SSTIs in inmates.^25^ The spread of epidemic strains causing recurrent SSTIs may be related to the PVL toxin, although no significant associations have been reported between illness severity or dissemination potential and presence of PVL genes.^26^^,^ 27 The present outbreak constitutes additional evidence that PVL-positive MSSA strains can cause recurrent invasive infections, in contrast to PVL-negative MSSA strains. The epidemic strain caused a large number of SSTIs during a short period compared to the number of sporadic SSTIs caused by PVL-negative MSSA before and after the outbreak.

In conclusion, this investigation is probably the first reported outbreak of PVL-positive MSSA SSTIs in a French prison. Challenges in controlling the outbreak included the absence of routine screening for MSSA carriage and high risk of recurrent infection due to the characteristics of the population (low educational level, poor compliance with antibiotic treatments, and poor adherence to hygiene measures leading to re-contamination with their own strain or cross-transmission in collective areas). The experience acquired during the outbreak helped to inform the 2012 French national guidelines issued by the French National Authority for Health and Ministry of Justice. These guidelines and those issued in 2011 by the Health Protection Agency in the UK should help to control communicable diseases in correctional facilities. Inmates should be able to receive tattoos under safety conditions identical to those available to the general public. Further investigations are needed to evaluate the risk of *S. aureus *transmission by tattooing in inmates.

## Competing Interests

The authors have declared that no competing interests exist.

## References

[1] Wertheim HF, Melles DC, Vos MC, van Leeuwen W, van Belkum A, Verbrugh HA, Nouwen JL. The role of nasal carriage in Staphylococcus aureus infections. Lancet Infect Dis. 2005 Dec;5(12):751-62. PubMed PMID:16310147. 16310147 10.1016/S1473-3099(05)70295-4

[2] Vandenesch F, Naimi T, Enright MC, Lina G, Nimmo GR, Heffernan H, Liassine N, Bes M, Greenland T, Reverdy ME, Etienne J. Community-acquired methicillin-resistant Staphylococcus aureus carrying Panton-Valentine leukocidin genes: worldwide emergence. Emerg Infect Dis. 2003 Aug;9(8):978-84. PubMed PMID:12967497. 12967497 10.3201/eid0908.030089 PMC3020611

[3] Kaltsas A, Guh A, Mediavilla JR, Varshney AK, Robiou N, Gialanellia P, Henry M, Levi MH, Fries BC. Frequency of panton-valentine leukocidin-producing methicillin-sensitive Staphylococcus strains in patients with complicated skin and soft tissue infection in bronx, new york. J Clin Microbiol. 2011 Aug;49(8):2992-5. PubMed PMID:21653777. 21653777 10.1128/JCM.00704-11 PMC3147728

[4] Kreienbuehl L, Charbonney E, Eggimann P. Community-acquired necrotizing pneumonia due to methicillin-sensitive Staphylococcus aureus secreting Panton-Valentine leukocidin: a review of case reports. Ann Intensive Care. 2011 Dec 22;1(1):52. PubMed PMID:22192614. 22192614 10.1186/2110-5820-1-52 PMC3259061

[5] Rasigade JP, Laurent F, Lina G, Meugnier H, Bes M, Vandenesch F, Etienne J, Tristan A. Global distribution and evolution of Panton-Valentine leukocidin-positive methicillin-susceptible Staphylococcus aureus, 1981-2007. J Infect Dis. 2010 May 15;201(10):1589-97. PubMed PMID:20367458. 20367458 10.1086/652008

[6] Lee CJ, Sankaran S, Mukherjee DV, Apa ZL, Hafer CA, Wright L, Larson EL, Lowy FD. Staphylococcus aureus oropharyngeal carriage in a prison population. Clin Infect Dis. 2011 Mar 15;52(6):775-8. PubMed PMID:21367730. 21367730 10.1093/cid/cir026 PMC3049338

[7] Lowy FD, Aiello AE, Bhat M, Johnson-Lawrence VD, Lee MH, Burrell E, Wright LN, Vasquez G, Larson EL. Staphylococcus aureus colonization and infection in New York State prisons. J Infect Dis. 2007 Sep 15;196(6):911-8. PubMed PMID:17703423. 17703423 10.1086/520933

[8] Main CL, Jayaratne P, Haley A, Rutherford C, Smaill F, Fisman DN. Outbreaks of infection caused by community-acquired methicillin-resistant Staphylococcus aureus in a Canadian correctional facility. Can J Infect Dis Med Microbiol. 2005 Nov;16(6):343-8. PubMed PMID:18159517. 18159517 10.1155/2005/698181 PMC2094999

[9] Pan ES, Diep BA, Carleton HA, Charlebois ED, Sensabaugh GF, Haller BL, Perdreau-Remington F. Increasing prevalence of methicillin-resistant Staphylococcus aureus infection in California jails. Clin Infect Dis. 2003 Nov 15;37(10):1384-8. PubMed PMID:14583874. 14583874 10.1086/379019

[10] Lepelletier D, Corvec S, Caillon J, Reynaud A, Rozé JC, Gras-Leguen C. Eradication of methicillin-resistant Staphylococcus aureus in a neonatal intensive care unit: which measures for which success? Am J Infect Control. 2009 Apr;37(3):195-200. PubMed PMID:19181423. 19181423 10.1016/j.ajic.2008.09.024

[11] Jarraud S, Mougel C, Thioulouse J, Lina G, Meugnier H, Forey F, Nesme X, Etienne J, Vandenesch F. Relationships between Staphylococcus aureus genetic background, virulence factors, agr groups (alleles), and human disease. Infect Immun. 2002 Feb;70(2):631-41. PubMed PMID:11796592. 11796592 10.1128/IAI.70.2.631-641.2002 PMC127674

[12] Tenover FC, Arbeit RD, Goering RV, Mickelsen PA, Murray BE, Persing DH, Swaminathan B. Interpreting chromosomal DNA restriction patterns produced by pulsed-field gel electrophoresis: criteria for bacterial strain typing. J Clin Microbiol. 1995 Sep;33(9):2233-9. PubMed PMID:7494007. 7494007 10.1128/jcm.33.9.2233-2239.1995 PMC228385

[13] Rotily M, Delorme C, Rousseau S. Epidémiologie de l'infection à VIH et des hépatites en milieu carcéral: une étude multicentrique européenne. In: Recueil des actes. 1er congrès des médecins exerçant en milieu pénitentiaire. Nantes 20 and 21 March 1997. [cited 2012, July 9].

[14] Maree CL, Eells SJ, Tan J, Bancroft EA, Malek M, Harawa NT, Lewis MJ, Santana E, Miller LG. Risk factors for infection and colonization with community-associated methicillin-resistant Staphylococcus aureus in the Los Angeles County jail: a case-control study. Clin Infect Dis. 2010 Dec 1;51(11):1248-57. PubMed PMID:21034197. 21034197 10.1086/657067 PMC3697429

[15] Kluger N. [Cutaneous infections related to permanent tattooing]. Med Mal Infect. 2011 Mar;41(3):115-22. PubMed PMID:21144685. 21144685 10.1016/j.medmal.2010.09.013

[16] Hellard ME, Aitken CK, Hocking JS. Tattooing in prisons--not such a pretty picture. Am J Infect Control. 2007 Sep;35(7):477-80. PubMed PMID:17765561. 17765561 10.1016/j.ajic.2006.08.002

[17] Abiona TC, Balogun JA, Adefuye AS, Sloan PE. Body art practices among inmates: Implications for transmission of bloodborne infections. Am J Infect Control. 2010 Mar;38(2):121-9. PubMed PMID:19822379. 19822379 10.1016/j.ajic.2009.06.006

[18] Ministère de la Santé et Ministère de la Justice et des Libertés. Politique de santé pour les personnes placées sous mains de justice. Plan d'actions stratégiques 2010-2014 [cited 2012, April 24].

[19] Health Protection Agency. Prevention of infection and communicable disease control in prisons and places of detention. July 2011 [cited 2012, April 24].

[20] Bick JA. Infection control in jails and prisons. Clin Infect Dis. 2007 Oct 15;45(8):1047-55. PubMed PMID:17879924. 17879924 10.1086/521910

[21] Aiello AE, Lowy FD, Wright LN, Larson EL. Meticillin-resistant Staphylococcus aureus among US prisoners and military personnel: review and recommendations for future studies. Lancet Infect Dis. 2006 Jun;6(6):335-41. PubMed PMID:16728319. 16728319 10.1016/S1473-3099(06)70491-1

[22] Methicillin-resistant Staphylococcus aureus skin or soft tissue infections in a state prison--Mississippi, 2000. MMWR Morb Mortal Wkly Rep. 2001 Oct 26;50(42):919-22. PubMed PMID:11699844. 11699844

[23] Methicillin-resistant Staphylococcus aureus infections in correctional facilities---Georgia, California, and Texas, 2001-2003. MMWR Morb Mortal Wkly Rep. 2003 Oct 17;52(41):992-6. PubMed PMID:14561958. 14561958

[24] From the Centers for Disease Control and Prevention. Methicillin-resistant Staphylococcus aureus skin or soft tissue infections in a state prison--Mississippi, 2000. JAMA. 2002 Jan 9;287(2):181-2. PubMed PMID:11799962. 11799962

[25] David MZ, Mennella C, Mansour M, Boyle-Vavra S, Daum RS. Predominance of methicillin-resistant Staphylococcus aureus among pathogens causing skin and soft tissue infections in a large urban jail: risk factors and recurrence rates. J Clin Microbiol. 2008 Oct;46(10):3222-7. PubMed PMID:18685002. 18685002 10.1128/JCM.01423-08 PMC2566069

[26] Lina G, Piémont Y, Godail-Gamot F, Bes M, Peter MO, Gauduchon V, Vandenesch F, Etienne J. Involvement of Panton-Valentine leukocidin-producing Staphylococcus aureus in primary skin infections and pneumonia. Clin Infect Dis. 1999 Nov;29(5):1128-32. PubMed PMID:10524952. 10524952 10.1086/313461

[27] Voyich JM, Otto M, Mathema B, Braughton KR, Whitney AR, Welty D, Long RD, Dorward DW, Gardner DJ, Lina G, Kreiswirth BN, DeLeo FR. Is Panton-Valentine leukocidin the major virulence determinant in community-associated methicillin-resistant Staphylococcus aureus disease? J Infect Dis. 2006 Dec 15;194(12):1761-70. PubMed PMID:17109350. 17109350 10.1086/509506

